# ﻿*Ghatippuspaschima*, a new species and genus of plexippine jumping spider from the Western Ghats of India (Salticidae, Plexippini, Plexippina)

**DOI:** 10.3897/zookeys.1191.114117

**Published:** 2024-02-12

**Authors:** Kiran Marathe, Wayne P. Maddison, Krushnamegh Kunte

**Affiliations:** 1 Department of Zoology, University of British Columbia, 6270 University Boulevard, Vancouver, British Columbia, V6T 1Z4, Canada; 2 National Centre for Biological Sciences, Tata Institute of Fundamental Research, GKVK Campus, Bengaluru, 560065, India; 3 Departments of Zoology and Botany and Beaty Biodiversity Museum, University of British Columbia, 6270 University Boulevard, Vancouver, British Columbia, V6T 1Z4, Canada

**Keywords:** Araneae, biodiversity research, classification, phylogenomics, systematics, taxonomy

## Abstract

We propose a new genus of plexippine jumping spiders from the Western Ghats of India based on the new species *Ghatippuspaschima***gen. et sp. nov.** While it bears a superficial resemblance to *Pancorius* in body form and *Hyllus* in membrane-bearing embolus, our UCE phylogenomic data—the first to resolve broad relationships within the Plexippina—as well as morphological features justify its status as a new genus. In addition to the molecular data and morphological descriptions, we provide photographs of living specimens of *Ghatippuspaschima***gen. et sp. nov.** and information on their natural history.

## ﻿Introduction

The Western Ghats of India, one of the hottest hotspots of biodiversity, awaits more than chance-based reporting of salticid spider diversity. Systematic surveys may reveal previously undiscovered salticids critical to understanding the region’s ecosystems and the broader context of salticid diversity and phylogeny. Our 2019 surveys in a private estate in Kodagu, Karnataka, for instance, uncovered one such salticid lineage, of the subtribe Plexippina. Here, we describe that new species and propose a new genus for it based on phylogenomic evidence and morphology.

The subtribe Plexippina (Salticinae, Plexippini), an Old World group except for two New World species of *Evarcha* Simon, 1902, is species-rich, containing over 500 described species currently placed in 37 genera worldwide (Maddison 2015; Metzner 2023; World Spider Catalog 2023). Their combination of high diversity, conservative body forms, and simple genitalia have hindered the discovery of synapomorphies that could delimit genera, making the group taxonomically challenging. Placing new species in genera without evidence explicitly stated and interpreted phylogenetically has led to decisions about generic divisions (e.g. Prószyński’s 2018 splitting of *Evarcha*) that are weakly supported and sometimes not broadly accepted (Kropf et al. 2019; World Spider Catalog 2023). Despite the taxonomic mess within the subtribe, what species are included in the Plexippina has remained more or less stable based on a combination of morphological (Maddison 1996, 2015) and molecular data (Maddison and Hedin 2003; Maddison et al. 2008; Bodner and Maddison 2012).

The first steps to our modern concept of Plexippina were taken by Maddison (1996), based on the form of the male endite’s serrula and the palp. Molecular data subsequently showed that some of the genera he included (e.g. *Sibianor* Logunov, 2001) are instead harmochirines (Maddison and Hedin 2003; Maddison et al. 2008; Bodner and Maddison 2012), leading to Maddison’s (2015) refined concept of the Plexippina. Using these studies as context, we here examine phylogenomically the relationships of the newly discovered plexippine lineage from the Western Ghats. Its placement would be unclear by morphology alone, as it is morphologically similar to *Hyllus* C.L. Koch, 1846 in male genitalia and *Pancorius* Simon, 1902 in its body form.

In the course of this work, we provide the first-ever plexippine phylogenomic tree, based on ultraconserved element data (Faircloth 2017; Zhang et al. 2023), contributing to further understanding of the relationship among plexippine genera and salticids in general (see Maddison et al. 2020a, b).

## ﻿Materials and methods

### ﻿Materials examined

The Indian specimens examined in this study are deposited in the Biodiversity Lab Research Collections of the National Centre for Biological Sciences (NCBS), Bengaluru, India (http://biodiversitycollections.in/). Individual specimens are identified by three-digit voucher codes prefixed with “**IBC-BP**” and “**IBC-BX**”; in addition, some are also identified by code numbers starting “**AS19.**”. Non-Indian specimens are deposited in the University of British Columbia Spencer Entomological Collection. Codes beginning with “**WPM#19**-” indicate a collecting event of location and date, and thus may apply to more than one specimen.

### ﻿Morphology

A drawing tube attached to a Nikon ME600L compound microscope was used to prepare illustrations. Clove oil was used for clear viewing of epigyna after digesting the internal epigynal soft tissues with pancreatin. Preserved specimens were photographed using an Olympus OM-D E-M10 II mounted on an Olympus SZX12 stereoscope (for bodies) and a Nikon D7000 mounted on a Nikon ME600L compound microscope (for copulatory organs). Photographs were stacked using Helicon Focus 8.2.1 Pro. Living specimens were photographed with an Olympus OM-D E-M10 II camera with a 60 mm macro lens.

Descriptions are based on ethanol-preserved specimens. The descriptions were written with primary reference to the focal specimen indicated, which was used for measurements and carefully checked for details, but they apply as far as known to the other specimens examined. Carapace length was measured from the anterior base of the median eyes to the posterior margin of the carapace. The abdomen was measured from its anterior edge to the posterior end of the anal tubercle. All the measurements are in millimetres. Leg measurements are represented as follows: total length (femur, patella, tibia, metatarsus, and tarsus). Abbreviations used here are as follows: **ALE**, anterior lateral eye; **AME**, anterior median eye; **PME**, posterior median eye; **PLE**, posterior lateral eye; **RTA**, retrolateral tibial apophysis.

### ﻿Taxon sampling for phylogenomics

The set of 18 species (15 ingroup and 3 outgroup species) used in the phylogenomic analysis, and with their taxonomic authority indicated, is listed in Table 1. The selection of ingroup taxa was determined based on the limits of Plexippina, informed by previous phylogenetic studies (Maddison et al. 2008; Bodner and Maddison 2012) and synthesis work by Maddison (2015). The taxon sampling strategy aimed to maximize the representation of plexippine genera and their morphological diversity, including those most similar and relatively least similar to the focal species of this work. The two genera viewed as morphologically most similar to the new species, and thus candidate genera to contain it, are *Hyllus* and *Pancorius*. Thus, two distinct species of each of those were included to give them the best chance of linking to the new species. Otherwise, 11 other plexippine genera representing diverse body forms were included, for a total of 15 ingroup taxa representing 13 genera. These 13 ingroup genera represent ~86% of the plexippine genera known from India. The selection of outgroup taxa, two harmochirines and one salticine, was based on previous salticid phylogenetic studies (Maddison et al. 2008, 2014, 2017; Bodner and Maddison 2012; Maddison 2015).

**Table 1.** Specimens used in phylogenomic analysis.

Species	Voucher	Sex	Locality	GPS coordinates (lat., long.)
*Anarrhotusfossulatus* Simon, 1902	AS19.1319	♂	Singapore	1.379, 103.816
*Artabruserythrocephalus* (C.L. Koch, 1846)	AS19.2205	♂	Singapore	1.355–7, 103.774–5
*Baryphasahenus* Simon, 1902	d536	♂	South Africa	-25.95, 30.56
*Bianormaculatus* (Keyserling, 1883)	NZ19.9864	♂	New Zealand	-42.1691, 172.8090
*Carrhotus* sp.	AS19.4650	♂	India	12.2145, 75.653–4
*Epeus* sp.	DDKM21.055	♂	Singapore	1.355, 103.78
*Evacinbulbosa* (Żabka, 1985)	AS19.2123	♂	Singapore	1.406, 103.971
*Evarchafalcata* (Clerck, 1757)	RU18-5264	♂	Russia	53.721, 77.726
*Ghatippuspaschima* Marathe & Maddison sp. nov.	IBC-BP833/ AS19.3805	♂	India	12.220–1, 75.657–8
*Habronattushirsutus* (G.W. Peckham & E.G. Peckham, 1888)	IDWM.21018	♂	Canada	48.827, -123.265
*Hylluskeratodes* (van Hasselt, 1882)	DDKM21.028	♂	Malaysia	3.325, 101.753
*Hyllussemicupreus* (Simon, 1885)	AS19.4415	♂	India	12.2156, 75.6606
*Pancoriusdentichelis* (Simon, 1899)	SWK12-0042	♂	Malaysia	1.605–6, 110.185–7
*Pancoriuspetoti* Prószyński & Deeleman-Reinhold, 2013	SWK12-0195	♂	Malaysia	1.603–4, 110.185
*Plexippuspaykulli* (Audouin, 1826)	AS19.7337	♂	India	12.825–6, 78.252–3
*Ptocasiusweyersi* Simon, 1885	DDKM21.069	♂	Singapore	1.36, 103.78
*Telamoniafestiva* Thorell, 1887	DDKM21.048	♂	China	21.8105, 107.2925
*Thyeneimperialis* (Rossi, 1846)	AS19.6443	♂	India	12.216, 76.625

### ﻿Ultraconserved element (UCE) data

Molecular data was gathered for UCE loci using target enrichment sequencing methods (Faircloth 2017). One to four legs were used for DNA extraction using the Qiagen DNeasy Blood and Tissue Kit following the manufacturer protocol. The quality and quantity of the genomic DNA was measured using a NanoDrop 200c Spectrophotometer. For the target enrichment UCE sequencing, dual-indexed TruSeq-style libraries were prepared following methods used previously (e.g. Maddison et al. 2020b). Targeted enrichment using the RTA_v2 probeset (Zhang et al. 2023) was performed using the myBaits v. 4.01 protocol (Arbor Biosciences, https://arborbiosci.com/wp-content/uploads/2023/06/myBaits_Manual_v5.03.pdf). Libraries were sequenced on partial lanes of illumina NovaSeq 6000 S4 runs with 150-bp paired end reads.

Raw demultiplexed reads were processed with PHYLUCE v. 1.6 (Faircloth 2016), quality control and adapter removal were performed with Illumiprocessor wrapper (Faircloth 2013), and assemblies were created with SPAdes v. 3.14.1 (Nurk et al. 2013) using options at default settings. The UCE loci were recovered using RTA_v2 probeset (Zhang et al. 2023). The recovered loci were aligned with MAFFT using L-INS-i option (Katoh and Standley 2013). The aligned UCE loci were then trimmed with Gblocks (Castresana 2000; Talavera and Castresana 2007) using –b1 0.5, –b2 0.7, –b3 8, –b4 8, –b5 0.4 setting and re-aligned with MAFFT using L-INS-i option within Mesquite v. 3.61 (Maddison and Maddison 2019). As in the analysis of Maddison et al. (2020a), suspected paralogous loci were deleted based on branch lengths in RAxML (Stamatakis 2014) inferred gene trees. Loci represented in fewer than 10 taxa total were deleted.

### ﻿Phylogenetic analysis

Maximum-likelihood phylogenetic and bootstrap analyses were performed with IQ-TREE v. 1.6.12 (Nguyen et al. 2015) using the Zephyr v. 3.1 package (Maddison and Maddison 2020) in Mesquite v. 3.61 (Maddison and Maddison 2019) on the concatenated, unpartitioned UCE dataset with 15 ingroup and three outgroup taxa. For the phylogenetic tree inference, the option -m TEST (standard model selection followed by tree inference, edge-linked partition model, no partition-specific rates) was used with 10 search replicates. For the bootstrap analysis, the same option as the tree inference was used with 1000 search replicates.

### Data availability

The raw sequence reads obtained from UCE capture are stored within the Sequence Read Archive (BioProject: PRJNA1067139, https://www.ncbi.nlm.nih.gov/bioproject/PRJNA1067139) and their accession numbers are listed in Table 1. The UCE loci matrices from SPAdes assemblies, pre-Gblocks, and the concatenated matrices used for phylogenetic and bootstrap analysis, along with trees, are available on the Dryad data repository (Link: https://doi.org/10.5061/dryad.zcrjdfnkw).

## ﻿Results

### ﻿Phylogenetic results

Table 2 lists the sequence data recovered from the 18 taxa. On average 2844 UCE loci per taxa (minimum 2255, maximum 3092) were initially recovered. Of these total loci, on average 2807 loci survived per taxa (min. 2225, max. 3054) after removing suspected paralogous loci based on branch lengths, and on average 2722 loci remained per taxa (min. 2205, max. 2956) after removing loci represented in fewer than 10 taxa. In total, 3060 UCE loci were represented in the resulting dataset, which were concatenated into the final matrix, in which each taxon had on average ~2.2 million base pairs of sequence data (min. 965482, max. 2414600).

**Table 2.** Specifics of molecular data used for this phylogenomic analysis. Molecular data was generated based on RTA_v2 probeset. “**SRA**” is Sequence Read Archive accession number available through NCBI; “**Reads pass QC**” is the number of reads after the removal of adapter-contamination and low-quality bases using Illumiprocessor; “**Total UCE loci**” is the total number of UCE loci recovered with RTA_v2 probeset; “**After paralogy filter**” is the number of UCE loci after deletion of suspected paralogous loci based on branch length ratios; “**In at least 10 taxa**” is the number of UCE loci in at least 10 or more taxa after branch length criteria; “**Filtered UCE sequence length**” is the concatenated sequence length of filtered UCE loci; “**Total loci**” is the number of UCE loci represented among all taxa.

Species	Voucher	SRA	Reads pass QC	Total UCE loci	After paralogy filter	In at least 10 taxa	Filtered UCE sequence length
*Anarrhotusfossulatus*	AS19.1319	SRR27728361	15542927	2525	2492	2384	2057818
*Artabruserythrocephalus*	AS19.2205	SRR27728359	14903498	2837	2800	2736	2287255
*Baryphasahenus*	d536	SRR27728358	2653688	2255	2225	2205	965482
*Bianormaculatus*	NZ19.9864	SRR27728369	7914005	2954	2916	2794	2376468
*Carrhotus* sp.	AS19.4650	SRR27728370	5272657	2914	2877	2783	2284451
*Epeus* sp.	DDKM21.055	SRR27728357	13896435	2896	2859	2779	2403857
*Evacinbulbosa*	AS19.2123	SRR27728356	10851810	2765	2731	2628	2113380
*Evarchafalcata*	RU18-5264	SRR27728355	11538276	2761	2723	2659	2174281
*Ghatippuspaschima* sp. nov.	IBC-BP833/ AS19.3805	SRR27728354	7881860	2892	2854	2779	2381949
*Habronattushirsutus*	IDWM.21018	SRR27728360	6581974	2817	2784	2682	2187694
*Hylluskeratodes*	DDKM21.028	SRR27728353	11349372	2925	2886	2788	2367864
*Hyllussemicupreus*	AS19.4415	SRR27728368	9874003	2939	2905	2820	2377271
*Pancoriusdentichelis*	SWK12-0042	SRR27728367	6025337	3092	3054	2956	2251455
*Pancoriuspetoti*	SWK12-0195	SRR27728366	5116119	2980	2943	2853	2245013
*Plexippuspaykulli*	AS19.7337	SRR27728365	7445183	2930	2892	2799	2139754
*Ptocasiusweyersi*	DDKM21.069	SRR27728364	9926900	2878	2840	2768	2279296
*Telamoniafestiva*	DDKM21.048	SRR27728363	7908436	2948	2911	2831	2414600
*Thyeneimperialis*	AS19.6443	SRR27728362	7797854	2888	2851	2763	2371167
			Average:	2844.2	2807.9	2722.6	2204391.9
			Minimum:	2255	2225	2205	965482
			Maximum:	3092	3054	2956	2414600
			Total loci:	3377	3335	3060	

**Figure 1.**
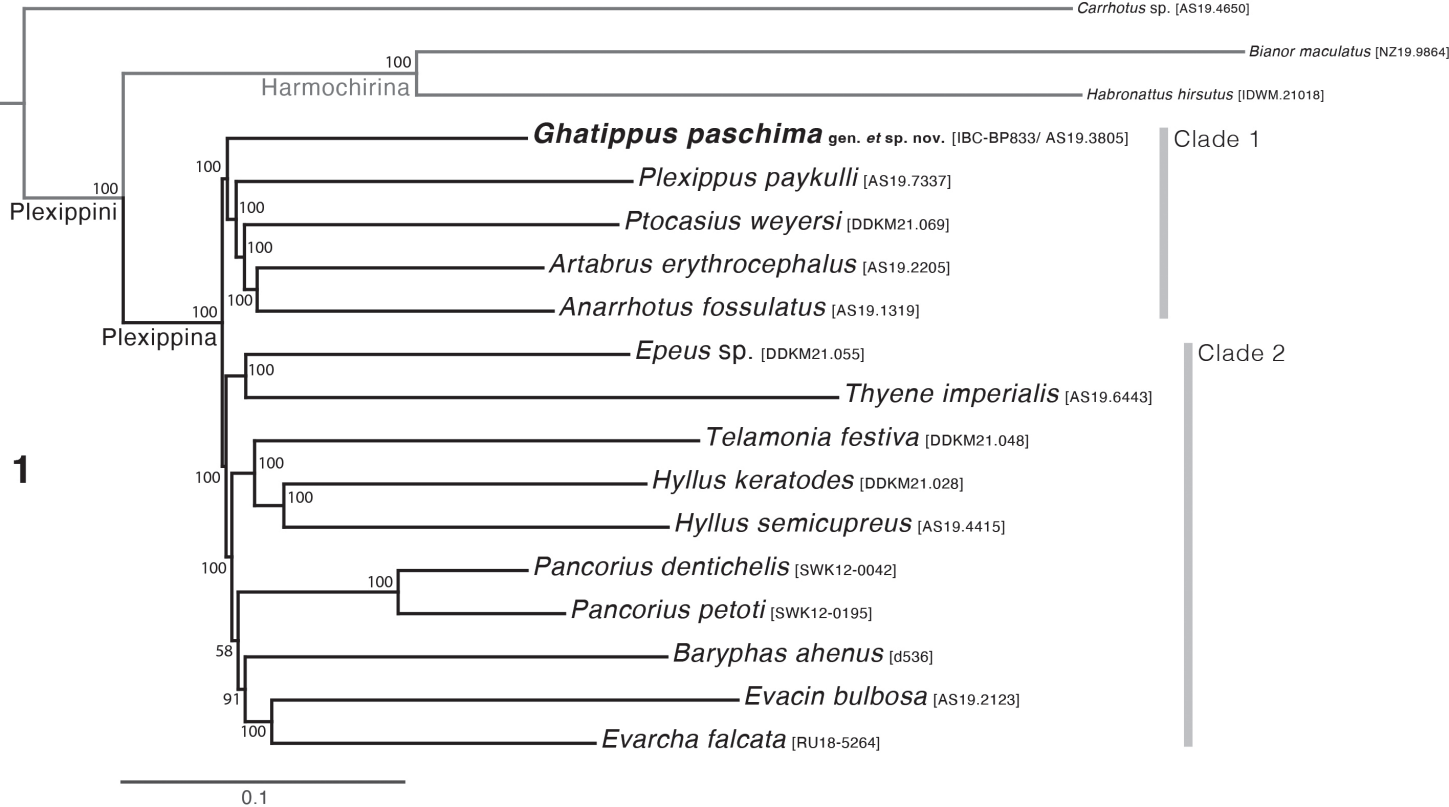
Maximum-likelihood tree, best tree of 10 replicates inferred using IQ-TREE, from concatenated dataset of 3060 ultraconserved element loci. Numbers at the nodes are percentage of 1000 bootstrap replicates recovering the clade. *Ghatippuspaschima* sp. nov. is recovered distantly (see Clade 1) from morphologically similar *Hyllus* and *Pancorius* (see Clade 2).

The phylogenetic results are shown in Fig. 1. The subtribes Plexippina and Harmochirina are recovered as reciprocally monophyletic, consistent with the previous phylogenetic studies with much less sequence data (Maddison and Hedin 2003; Maddison et al. 2008; Bodner and Maddison 2012). Within the Plexippina, two major clades are recognized (marked in Fig. 1). Bootstrap values are generally high, showing that the relationships are well supported, as might be expected with this volume of sequence data.

*Ghatippus* gen. nov. is recovered as sister to all the genera in clade 1 (see Fig. 1): (*Ghatippus*, (*Plexippus*, (*Ptocasius*, (*Anarrhotus*, *Artabrus*)))). This phylogenetic position of *Ghatippus* gen. nov. necessitates its recognition as a new genus. Any other taxonomic decision apart from creating a new genus, whether to include it in a phylogenetically closely related genus or in another morphologically similar plexippine genus, would render the genus in which it is placed either paraphyletic or polyphyletic. The only other phylogenetically meaningful option, besides creating a new genus, would be to lump all the genera in clade 1 into a single genus. This would generate a massive genus of highly diverse body forms that would go against all traditions of salticid generic limits. A far better choice is to recognize *Ghatippus* gen. nov. as a new genus.

The choice to establish a new genus is further substantiated by morphology. Within clade 1, *Ghatippus* gen. nov. is unique with its membrane bearing medium-long embolus. In contrast, *Anarrhotus* Simon, 1902 and *Plexippus* C.L. Koch, 1846 have a short embolus, while *Artabrus* Simon, 1902 and *Ptocasius* Simon, 1885 have a medium to long, thin embolus. Importantly, all four of these lack a membrane-bearing embolus.

### ﻿Taxonomic results

**Family Salticidae Blackwall, 1841**

**Tribe Plexippini Simon, 1901**

**Subtribe Plexippina Simon, 1901**

#### ﻿ Ghatippus

Taxon classificationAnimaliaAraneaeSalticidae

Marathe & Maddison gen. nov.

7CA59091-BA8E-59FE-A0C0-A700A57773D9

https://zoobank.org/1E8E60B3-FBE6-4DB5-83B0-38BFFE401862

Figs 2–5 , 6, 7 , 8–17 , 18–25 , 26–40

##### Type species.

*Ghatippuspaschima* Marathe & Maddison, sp. nov.; by monotypy.

##### Etymology.

The generic name *Ghatippus* gen. nov. combines the word ‘Ghat’, representing the collecting locality—the Western Ghats Mountain range—with the distinctive suffix found in several plexippine genera. The generic name is assigned to the masculine gender.

##### Diagnosis.

**Figures 2–5.**
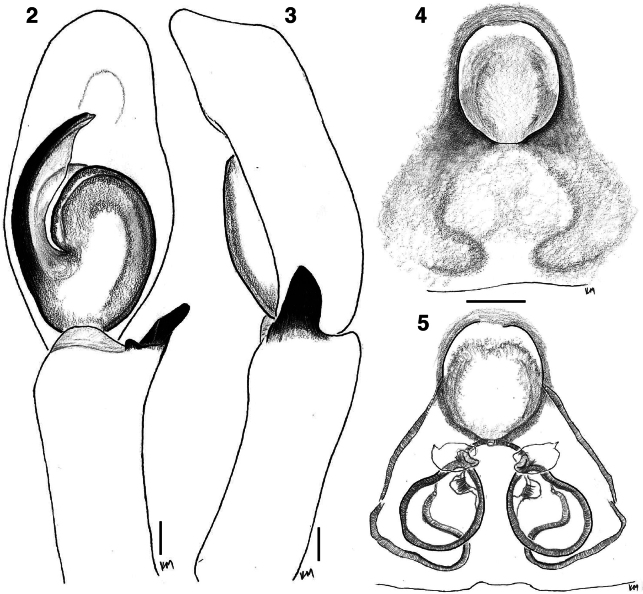
*Ghatippuspaschima* sp. nov. genitalia **2** male left palp, ventral view (holotype IBC-BP817) **3** ditto, retrolateral view (holotype IBC-BP817) **4** epigyne, ventral view (paratype IBC-BP818) **5** vulva, dorsal view (paratype IBC-BP818). Scale bars: 0.1 mm.

**Figures 6, 7.**
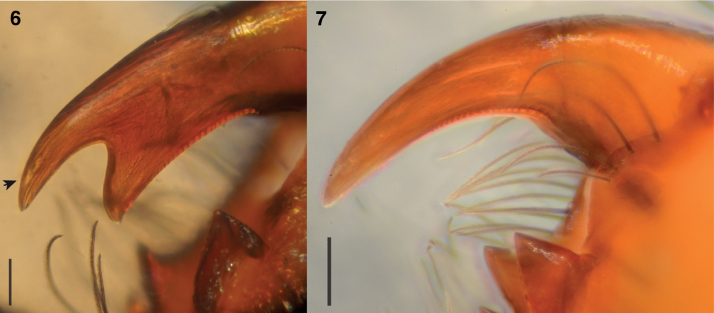
*Ghatippuspaschima* sp. nov., dorsal view of left chelicerae **6** paratype male, IBP-BP819 **7** paratype female, IBC-BP820 (arrow points to the true tip on the male chelicera bearing the venom duct). Scale bars: 0.1 mm.

The UCE phylogeny implies genetic diagnosability of *Ghatippus* gen. nov., but here we focus on the morphological distinctions. The membranous retrolateral edge of the embolus (Figs 2, 18) and lack of distinct epigynal coupling pockets (Figs 4, 20) differentiate *Ghatippus* gen. nov. from all members of clade 1 (Fig. 1) and other plexippines except *Hyllus*, *Thyene* Simon, 1885, and *Vailimia* Kammerer, 2006. Also, *Ghatippus* gen. nov. is the only plexippine reported to have a bifurcated male fang with nearly co-equal branch points (Figs 6, 12).

From *Hyllus*, *Ghatippus* gen. nov. differs in carapace (higher, box-shaped, PLEs on tubercles in *Ghatippus* gen. nov. vs relatively lower, rounder, no tubercles in *Hyllus*), RTA (simple, short vs serrated, wide), cymbium (laterally narrow with a narrow apex vs robust, laterally wide with a broader apex), and copulatory ducts (short vs long). From *Thyene*, *Ghatippus* gen. nov. differs in embolus length (medium in *Ghatippus* gen. nov. vs long and coiled in *Thyene*), copulatory ducts (short vs long), and carapace (higher, box-shaped, PLEs on tubercles vs relatively lower, rounder, no tubercles). From *Vailimia*, *Ghatippus* gen. nov. differs in embolus length (medium in *Ghatippus* gen. nov. vs long in *Vailimia*), RTA (simple, short vs curvy, long), and spermathecae (simple vs globular). *Ghatippus* gen. nov. also has an oval abdomen and open posture typical for salticids, unlike *Vailimia*’s pointed abdomen and unusual stance, holding the legs close to the body in a compact crouch.

*Ghatippus* gen. nov. is most likely to be confused with *Pancorius* because of the high, box-shaped carapace with PLEs on tubercles, but *Pancorius* lacks the membrane-bearing embolus and has distinct epigynal coupling pockets.

#### ﻿ Ghatippus paschima

Taxon classificationAnimaliaAraneaeSalticidae

Marathe & Maddison sp. nov.

26539105-8AD8-5EDF-AE9E-014065D33058

https://zoobank.org/FAD7F75C-B5B9-4B6B-ABF4-621D8073A3C5

Figs 2–5 , 6, 7 , 8–17 , 18–25 , 26–40

##### Type materials.

All from India: Karnataka: Kodagu: Yavakapadi, Honey Valley area and deposited in Biodiversity Lab Research Collections, NCBS. ***Holotype***: Male, IBC-BP817, 12.2202°N, 75.6581°E, 1190–1230 m elev., 24 June 2019, K. Marathe & W. Maddison, WPM#19-071. ***Paratypes***: 5 ♂♂ and 5 ♀♀ (IBC-BP818 – IBC-BP827), data same as the holotype • 4 ♂♂ and 1 ♀ (IBC-BP828 – IBC-BP832), buildings and roadside, 12.22°N, 75.66°E, 1100 m elev., 23–28 June 2019, W. Maddison & K. Marathe, WPM#19-069 • 4 ♂♂ and 4 ♀♀ (IBC-BP833 – IBC-BP840), along stream, 12.220 to 12.221°N, 75.657 to 75.658°E, 1190 m elev., 24 June 2019, W. Maddison & K. Marathe, WPM#19-070 • 3 ♂♂ (IBC-BP841 – IBC-BP843), forest & grassland, 12.2156 to 12.2157°N, 75.6597 to 75.6606°E, 1300 m elev., 25 June 2019, W. Maddison & K. Marathe, WPM#19-075 • 2 ♂♂ (IBC-BP844 – IBC-BP845), forest & edge, 12.215 to 12.216°N, 75.659 to 75.661°E, 1300 m elev., 25 June 2019, W. Maddison & K. Marathe, WPM#19-077 • 1 ♀ (IBC-BP846), grassland, 12.2145°N, 75.653–75.654°E, 1280–1380 m elev., 26 June 2019, W. Maddison & K. Marathe, WPM#19-080 • 1 ♂ (IBC-BX501), Chingara Falls,12.232°N, 75.653°E, 970 m elev., 27 June 2019, Maddison/ Marathe/ Abhijith/ Pavan, WPM#19-084 • 1 ♂ (IBC-BX502), open woodland,12.216°N, 75.661°E, 1320 m elev., 28 June 2019, K. Marathe & W. Maddison, WPM#19-088.

**Figures 8–17.**
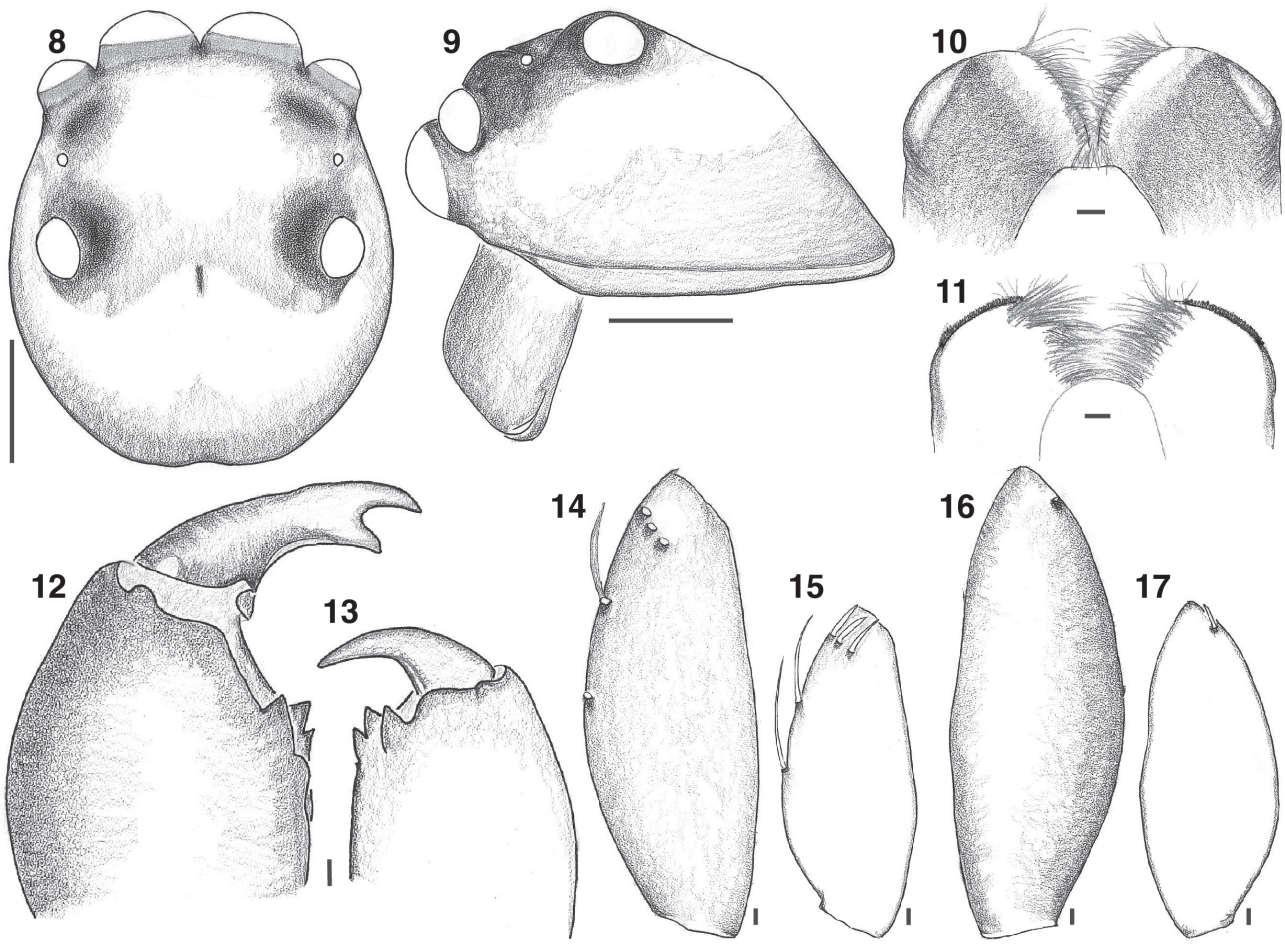
*Ghatippuspaschima* sp. nov. **8** male (paratype IBC-BP819) carapace, dorsal view **9** ditto, side view **10, 11** male endite, ventral and dorsal view respectively (paratype IBC-BP819) **12** male right chelicera, dorsal view (paratype IBC-BP819) **13** female left chelicera, dorsal view (paratype IBC-BP820) **14** male left femur of leg I, prolateral view (paratype IBC-BP819) **15** female left femur of leg I, prolateral view (paratype IBC-BP820) **16** male left femur of leg I, retrolateral view (paratype IBC-BP819) **17** female left femur of leg I, retrolateral view (paratype IBC-BP820). Scale bars: 1.0 mm (**6, 7**); 0.1 mm (**8–15**).

##### Etymology.

The specific epithet *paschima*, a noun in apposition, means “west” in both Sanskrit and Kannada.

##### Diagnosis.

As there is only one species in the genus, see the generic diagnosis.

##### Description.

**Figures 18–25.**
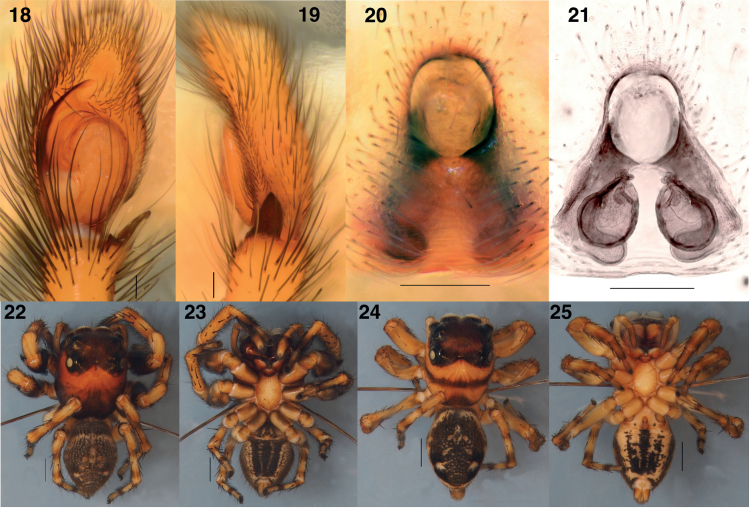
*Ghatippuspaschima* sp. nov. genitalia (top row) and alcohol preserved types habitus (bottom row) **18** male (holotype IBC-BP817) left palp, ventral view **19** ditto, retrolateral view **20** epigyne, ventral view (paratype IBC-BP818) **21** vulva, dorsal view (paratype IBC-BP818) **22** male (holotype IBC-BP817), dorsal view **23** ditto, ventral view **24** female (paratype IBC-BP818), dorsal view **25** ditto, ventral view. Scale bars: 0.1 mm for genitalia; 1.0 mm for bodies.

**Male** (focal specimen, holotype, IBC-BP817). ***Measurements***: Carapace 3.9 long, 3.3 wide. Abdomen 4 long, 2.5 wide. ***Leg measurements***: I–9.4 (3.1, 1.9, 2.3, 1.2, 0.9); II–6.9 (2.1, 1.6, 1.3, 1, 0.9); III–7.1 (2.6, 1.5, 1.7, 0.6, 0.7); IV–7.2 (2.2, 1.2, 1.7, 1.3, 0.8). Leg formula I-III-IV-II. ***Carapace*** mostly brown mottled with black. Ocular area dark brown, sparsely covered with lustrous yellowish-golden hairs. Distinct black bulge behind each ALE (Figs 8, 9, 22). Black around PMEs and PLEs. Thorax with steep slope, brown, sparsely covered with black hairs. Black along edges. ***Clypeus*** narrow, brown, covered in white hairs appearing like a moustache. ***Chelicerae*** dark brown. Vertical, about as wide as carapace, bulging. Fangs bifid, with second fork near true tip (bearing venom duct) and almost as long as tip (Figs 6, 12). ***Palp*** (Figs 2, 3, 18, 19) yellowish brown. Tibia about as long as patella. Relatively narrow cymbium. Medium-long embolus arising from base at about 7–8 o’clock. Retrolateral edge of embolus extended as firm transparent membrane. Simple kidney-bean-shaped tegulum, gently curved proximally. RTA short and wide blade, simple. ***Legs*** mostly yellowish, brownish near joints, generally robust. Femur I and II distinctively dark brown, robust, and stout, with vertical fringe of short black hairs dorsally and, near patella, posteriolaterally. Metatarsus I with ventral fringe of black hairs, and weaker fringe on metatarsus II. ***Abdomen*** ovoid, medium to dark brown, covered with scales that in life have golden or reddish sheen. Indistinct basal band paler, as are muscle attachment points and posterior medial chevron. Two distinct pale spots in posterior half, one on either side of chevron, and two smaller spots just in front of spinnerets. Spinnerets yellowish, covered with black hairs.

**Female** (focal specimen, paratype, IBC-BP818). ***Measurements***: Carapace 3.4 long, 2.8 wide. Abdomen 4.2 long, 2.4 wide. ***Leg measurements***: I–5.4 (1.7, 1.1, 1.2, 0.9, 0.5); II–4.9 (1.7, 0.8, 1.2, 0.8, 0.4); III–6.9 (2, 1.2, 1.5, 1.5, 0.7); IV–6.3 (1.7, 1, 1.5, 1.5, 0.6). Leg formula III-IV-I-II. ***Carapace*** yellow (thorax) to brown (head). Ocular area dark brown, sparsely covered with lustrous white hairs. Distinct black bulge behind each ALE. Black around PMEs and PLEs. Thorax with steep slope, yellowish brown, sparsely covered with black hairs. With origin near front, brown band encircles carapace close to transition between ocular area and thorax. Brown along edges. ***Clypeus*** narrow, brown, covered with white hairs but more sparsely than in male. ***Chelicerae*** yellowish brown. Vertical, narrower than extent of carapace, not bulging as in male, with simple unbifurcated fangs (Figs 7, 13). ***Legs*** mostly yellowish and some brown near joints. ***Abdomen*** ovoid, dark brown but with paler basal band (extended posteriorly to encircle the abdomen), muscle attachment points, and posterior medial chevron. On either of the chevron the brown is especially dark, almost black, and contains distinct pale spot (Fig. 24). ***Epigyne*** (Figs 4, 5, 20, 21): two crescent-shaped anterior copulatory openings share common atrium. No epigynal coupling pocket visible, though there is slight medial indentation of the epigastric furrow. Simple round spermathecae with flattened (lamellar) copulatory ducts ventrally. Fertilizations ducts broad, placed anteriorly on spermathecae.

**Figures 26–40.**
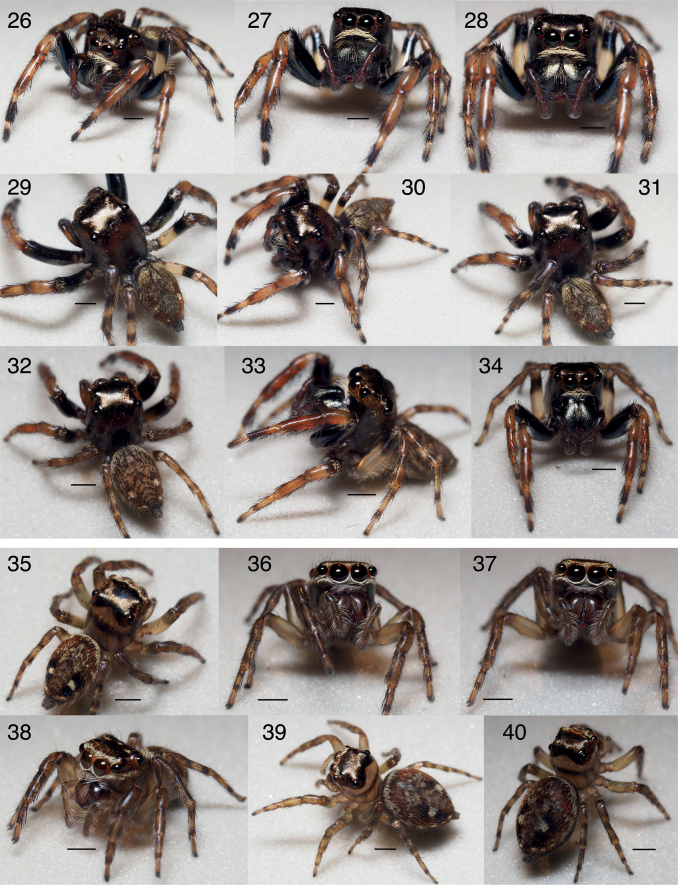
Habitus of *Ghatippuspaschima* sp. nov. **26–31** male (IBC-BP828/ AS19.4384) **32–34** male (IBC-BP833/ AS19.3805) **35–38** female, (IBC-BP834/ AS19.3814) **39, 40** (IBC-BP835/ AS19.3821). Scale bar: 1.0 mm.

##### Additional materials.

All from India: Kerala: near Thalappuzha, Fringe Ford, and deposited in Biodiversity Lab Research Collections, NCBS. 1 ♂ (IBC-BX503), forest path, 11.888°N, 75.692–75.963°E, 1020 m elev., 1 July 2019, W. Maddison & K. Marathe, WPM#19-095 • 1 ♀ (IBC-BX504), camp area, 11.884°N, 75.965°E, 990 m elev., 1–2 July 2019, W. Maddison & K. Marathe, WPM#19-099 • 3 ♂♂ and 1 ♀ (IBC-BX505 – IBC-BX508), forest, 11.88°N, 75.97°E, 1150 m elev., 2 July 2019, K. Marathe & W. Maddison, WPM#19-102.

##### Natural history.

*Ghatippuspaschima* sp. nov. was found commonly in both Kodagu and Kerala. Most collecting days in both locations were rainy and overcast. The spiders seemed to be exclusively vegetation dwellers, often found on small to medium-sized trees. Although they were collected from diverse habitats, they were mostly collected in the understorey, edge, and disturbed habitats of the evergreen forests of Honey Valley Estate in Kodagu. In Fringe Ford, Kerala, they were collected from the secondary evergreen growth of an inoperative tea estate.

While male and female salticids typically differ in colour, sexual dimorphism in the fangs is noteworthy. Male fangs are bifid, but female fangs are not (Figs 6, 7, 12, 13). The bifid fangs may possibly be used to hold females during mating, in male-to-male combat, or have a sex-limited ecological function.

##### Discussion.

Plexippines account for about ~9% of the total salticid diversity worldwide, with about ~8% of the world’s plexippine diversity documented in India (World Spider Catalog 2023). The 45 plexippine species previously known from India, out of 566 species worldwide, belong to 16 genera (Caleb 2019; World Spider Catalog 2023): *Anarrhotus* Simon, 1902 (1 sp. in India, of 2 worldwide), *Burmattus* Prószyński, 1992 (1 in India, of 5 worldwide), *Colopsus* Simon, 1902 (3 of 8), *Dexippus* Thorell, 1891 (3 of 4), *Epeus* G. W. Peckham & E. G. Peckham, 1886 (5 of 19), *Evarcha* Simon, 1902 (3 of 92), *Hyllus* C. L. Koch, 1846 (4 of 67), *Orientattus* Caleb, 2020 (1 of 4), *Pancorius* Simon, 1902 (9 of 45), *Plexippus* C. L. Koch, 1846 (4 of 42), *Pseudamycus* Simon, 1885 (1 of 10), *Ptocasius* Simon, 1885 (1 of 68), *Telamonia* Thorell, 1887 (3 of 40), *Thyene* Simon, 1885 (3 of 55), *Vailimia* Kammerer, 2006 (2 of 6), and *Yaginumaella* Prószyński, 1979 (1 of 14).

While we are beginning to see a steady uptick in the number of new plexippines being described (World Spider Catalog 2023), the unique endemic lineages and their radiations in India are still largely unexplored. With the addition of *Ghatippuspaschima* sp. nov., potentially an endemic lineage, the number of plexippines stands at 46 species and 17 genera for India.

## Supplementary Material

XML Treatment for Ghatippus

XML Treatment for Ghatippus paschima

## ﻿Additional information

### Author ORCIDs

Kiran Marathe https://orcid.org/0000-0002-7364-3475

Wayne P. Maddison https://orcid.org/0000-0003-4953-4575

Krushnamegh Kunte https://orcid.org/0000-0002-3860-6118

### Data availability

All of the data that support the findings of this study are available in the main text.
